# Genomics and Therapeutic Vulnerabilities of Primary Bone Tumors

**DOI:** 10.3390/cells9040968

**Published:** 2020-04-14

**Authors:** Katia Scotlandi, Claudia Maria Hattinger, Evelin Pellegrini, Marco Gambarotti, Massimo Serra

**Affiliations:** 1 Laboratory of Experimental Oncology, IRCCS Istituto Ortopedico Rizzoli, via di Barbiano 1/10, 40136 Bologna, Italy; claudia.hattinger@ior.it (C.M.H.); evelin.pellegrini@gmail.com (E.P.); 2 Department of Pathology, IRCCS Istituto Ortopedico Rizzoli, via di Barbiano 1/10, 40136 Bologna, Italy; marco.gambarotti@ior.it

**Keywords:** osteosarcoma, Ewing sarcoma, chondrosarcoma, genomics, personalized therapy

## Abstract

Osteosarcoma, Ewing sarcoma and chondrosarcoma are rare diseases but the most common primary tumors of bone. The genes directly involved in the sarcomagenesis, tumor progression and treatment responsiveness are not completely defined for these tumors, and the powerful discovery of genetic analysis is highly warranted in the view of improving the therapy and cure of patients. The review summarizes recent advances concerning the molecular and genetic background of these three neoplasms and, of their most common variants, highlights the putative therapeutic targets and the clinical trials that are presently active, and notes the fundamental issues that remain unanswered. In the era of personalized medicine, the rarity of sarcomas may not be the major obstacle, provided that each patient is studied extensively according to a road map that combines emerging genomic and functional approaches toward the selection of novel therapeutic strategies.

## 1. Introduction

Bone sarcomas are rare diseases, with an overall annual incidence of 1 case per 100,000 adults in Europe, and they represent 2% of all human neoplasms. They include diverse mesenchymal malignancies that arise from bone, cartilage and connective tissues, representing a very heterogenous group of tumors which range from indolent to very aggressive and metastatic. The World Health Organization (WHO) has recently reviewed the classification of sarcomas based on both the established histological features and molecular alterations [1]. From a molecular/genetics point of view, sarcomas have traditionally been classified into two major categories, the first including sarcomas with simple, near-diploid karyotypes and simple, translocation-associated alterations, and the second comprising tumors with complex and unbalanced karyotypes, characterized by multiple genomic aberrations. A detailed listing of the genetic features in sarcomas included in the two categories is available in the excellent previous reviews [2,3]. These categories, however, do not fully reflect the genetic diversity of the different tumors. The disposal of widespread genome- and epigenome-wide profiling has just started to reveal how heterogeneous sarcomas are at the molecular/genetic level and has resulted in the identification of reliable diagnostic and prognostic/predictive factors and novel guidance for medical treatments. So far, treatment of sarcomas has historically been rather similar for all the subtypes, comprising conventional chemotherapy, surgery and irradiation, and it is hoped that the application of this rapidly expanding knowledge not only refines diagnosis but also impacts the way in which clinical studies are designed and conducted. This review summarizes the current state-of-art molecular comprehension of primary bone tumors, and how this information is being translated into novel, more personalized, risk-based clinical studies. Particularly, the review highlights the molecular mechanisms of sarcomagenesis for the three most common malignant primary tumors of bone: osteosarcoma, Ewing sarcoma and chondrosarcoma, as paradigms of sarcomas typified by complex molecular alterations and genome instability, of translocation-associated sarcomas and of sarcomas whose natural histories’ include stages of tumor progression, respectively. The review also emphasizes the advances in genomics for a better understanding of sarcomagenesis, diagnosis and therapy of these tumors and notes the fundamental issues that remain unanswered.

## 2. Osteosarcoma

Osteosarcomas are malignant tumors composed of mesenchymal cells producing osteoid and immature bone. Based on their anatomo-clinical presentation, treatment and prognosis, high-grade varieties (90%) can be distinguished from low- and intermediate-grade varieties of osteosarcomas (10%) [4]. Most genetic studies have been performed on the so-called conventional high-grade osteosarcoma (HGOS), which is localized in the extremities, nonmetastatic at the clinical onset and arising in patients younger than 40 years.

**Figure 1:**
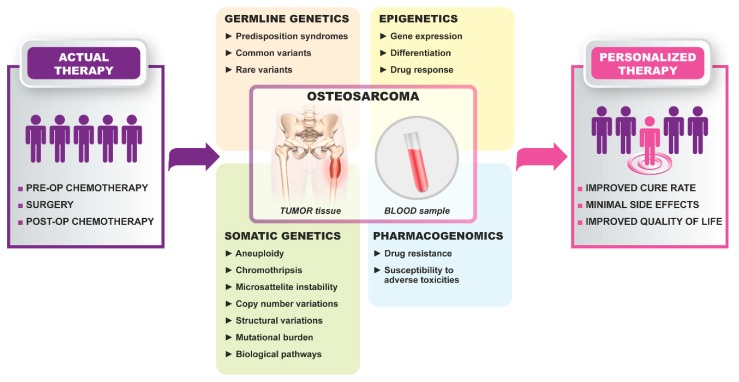
Transition toward personalized therapies in high-grade osteosarcoma.

Genetic characterization of HGOS has evolved during the last decade thanks to the integration of conventional and new generation candidate-driven and genome-wide technologies. The highly heterogeneous genetic background of HGOS opposes the identification of molecular and genetic biomarkers. However, several studies have highlighted candidate genetic markers, which can be translated into clinics in the near future (Figure 1), whereas other candidate targets have been recently considered to launch several clinical trials (Table 1).

### 2.1. Low-Grade Osteosarcomas

Low-grade osteosarcomas (parosteal and low-grade central osteosarcoma) and intermediate-grade osteosarcomas (periosteal) are very rare and grow slower than high-grade varieties of osteosarcomas. Removal of the tumor by surgery with wide margins is the gold standard of therapy. Prognosis is good unless a progression to a high-grade osteosarcoma occurs. Chemotherapy with the same drugs as used for HGOS is indicated only in patients with dedifferentiated parosteal osteosarcoma or low-grade central osteosarcoma progressed in HGOS [4].

Different to HGOS, on which the following sections will focus, low-grade osteosarcoma varieties (parosteal and low-grade central) frequently harbor amplifications of the 12q13-15 amplicon, including the MDM2 and CDK4 genes, as supernumerary ring or giant chromosomes. In a recent study of 22 low-grade osteosarcomas (3 low-grade central, 14 classic parosteal, and 5 dedifferentiated parosteal osteosarcomas), an MDM2 (12q15) amplification was revealed in 100% of the cases by fluorescence in situ hybridization. In addition, the fibroblast growth factor receptor substrate 2 (FRS2) gene at 12q15 was amplified in 95% of the cases but not in their histological mimics [5].

### 2.2. High-Grade Osteosarcomas

#### 2.2.1. Germline Genetics

Germline genetics have tried to identify common variants that may be associated with HGOS and to identify the genetic origin of osteosarcoma [6]. HGOS is associated with several rare and autosomal cancer predisposition syndromes, including the Li–Fraumeni syndrome (mutations of the TP53 gene), hereditary retinoblastoma (mutations of the RB1 gene), Bloom syndrome (mutations of the RECQL3 gene), Werner syndrome (mutations of the RECQL2 (WRN) gene), Rothmund–Thomson, Baller–Gerold and RAPADILINO syndromes (mutations of the RECQL4 gene). Candidate gene studies and, more recently, studies using high-throughput technologies like genome-wide association or next-generation sequencing (NGS) have been performed and have identified promising genes and pathways possibly involved in the aetiology of HGOS [6]. Additional rare variants were found in five cancer predisposition genes, APC, MSH2, PALB2, SQSTM1 and ERCC2, and in common variants in more than 20 genes belonging to bone homeostasis, growth, DNA repair-related, apoptosis, cell cycles, detox reactive oxidative species and tumor immunity pathways [6].

#### 2.2.2. Somatic Genetics

HGOS is characterized by a hyperdiploid, frequently a near-tri- to -tetraploid, an extremely complex karyotype displaying many copy number and structural aberrations with the result of losses and gains of the chromosomal fragments but without any pathognomonic chromosomal alteration [7]. The high genomic instability can be ascribed to chromothripsis (a massive genomic rearrangement due to a cataclysmic event in which chromosomes are fragmented and subsequently aberrantly assembled) and kataegis (high number of genetic changes due to localized hypermutation areas), both frequently described in HGOS [6,8]. A study focusing on somatic copy number alterations in 160 HGOS cases even indicated chromothripsis and near-tetraploidy associated with worse survival [9].

The results obtained in the studies using genome-wide NGS techniques have been discussed in two complete reviews [6,8]. Facing the genetic complexity and heterogeneity of HGOS, concomitantly with its rarity, is challenging. However, Rickel et al. provided a list of 53 candidate onco- and tumor-suppressor genes with validated somatic mutations identified in three studies [10,11,12]. All these genes belong to cancer signaling pathways regulating cell fate and survival and genome maintenance.

In order to better understand the genetic mechanisms of progression and evolution in HGOS, matched primary and normal samples from 13 pediatric patients with HGOS and matched metastatic or local recurrence samples of the same patients were analyzed by whole-genome sequencing and integrative analysis [13]. The data confirmed a highly heterogeneous mutational landscape including the presence of hypermutation and microsatellite instability. Recurrent somatic alterations were not found, except for structural variations (SVs) of TP53 in 9 out of 13 patients. Multiple germline alterations were found in 21 genes known to be implicated in cancer in 12 cases, but not of TP53. Copy number alterations showed higher conservation in metastases, whereas short variants were less conserved. The kinase insert domain receptor (KDR) gene was found to be commonly amplified and overexpressed in advanced cases and associated with a poor prognosis.

Given that HGOS has an average of 1.2 mutations per megabase, approximately 1000 samples are needed to obtain a complete catalog of the driver genes of intermediate frequencies. Results and their possible implications for the clinics have recently been reviewed and discussed [8,13].

#### 2.2.3. Candidate Predictive and Prognostic Biomarkers

Single nucleotide polymorphisms (SNPs) in several candidate genes of possible relevance for the biology, treatment response and drugs activation or detoxification of HGOS have been evaluated in order to indicate markers predictive of tumor progression, therapy response or patients’ susceptibility to develop treatment-related toxicities [14,15].

The candidate genes that have been most frequently studied in HGOS belong to DNA repair and methotrexate metabolism. However, it must be underlined that most studies were performed on a small patient series, and therefore the reported findings need to be further validated in a prospective way. Concerning DNA repair, the most consistent data have been reported for polymorphisms of the excision repair 1 (ERCC1) and excision repair 2 (ERCC2) genes, both involved in the nucleotide excision repair (NER) pathway. Although findings have sometimes been contradictory, two meta-analyses indicated that HGOS patients carrying the C allele of ERCC1 rs11615 [16,17] or the A allele of ERCC2 rs1799793 [17] have higher probabilities of responding to conventional chemotherapy among Chinese populations. Among genes related to methotrexate metabolism, the most widely studied in HGOS has been the 5,10-methylenetetrahydrofolate reductase (MTHFR) [15,18]. The most remarkable information on the possible clinical impact about this gene consisted with the association of the variant allele (T) of MTHFR rs1801133 with higher degrees of liver and hematologic toxicities in a series of 59 Han Chinese HGOS patients [19].

NGS approaches are also expected to provide insights leading to the identification of the HGOS driver genes, which may not consistently emerge with conventional techniques. The application of NGS and other genome-wide analyses to HGOS has further confirmed the presence of multiple genomic rearrangements, kataegis and a high degree of intratumor heterogeneity [10,20], but it has also indicated somatic gene mutations that appear to drive HGOS oncogenesis. One of the most clinically relevant information derived from the NGS and genome-wide studies applied to HGOS is the indication that the phosphatidylinositol 3-kinase/mammalian target of the rapamycin (PI3K/mTOR) pathway can be considered as a key target for HGOS treatment, providing the rationale for designing mTOR-targeting clinical trials [12,21,22] (Table 1). Phase I/II trials have been thus launched for patients with advanced soft-tissue and bone sarcomas (including HGOS) to assess the clinical efficacy of different mTOR inhibitors: temsirolimus (CCI-779, Torisel^®^, Wyeth Pharmaceuticals, Midrand, South Africa), everolimus (RAD-001; Novartis, Basel, Switzerland), deferolimus (AP23573, Ariad Pharmaceutical, Cambridge, MA, USA) and ridaforolimus (AP23573/MK-8669, Merck, Kenilworth, NJ). Although these drugs have shown encouraging antitumor activities and good tolerability, their therapeutic value for HGOS treatment still needs to be defined [12,21]. However, in a phase II trial performed by the Italian Sarcoma Group (ISG), the activity of everolimus in combination with sorafenib was evaluated in patients with inoperable HGOS, who progressed after conventional chemotherapy treatments. Despite that this study did not achieve the planned target of a 6-month progression-free survival in at least 50% of patients, 17 of the 38 enrolled patients did not present any tumor progression at 6 months, encouraging the use of this drug combination as a rescue treatment for patients with advanced or unresectable HGOS [23].

Another NGS finding with significant clinical relevance in HGOS, is the indication that this tumor frequently exhibits deficiencies in the BRCA1- and/or BRCA2-pathway (the so-called BRCAness phenotype). This body of information may constitute the basis for a future tailoring of HGOS treatment, since the BRCA1/2 pathway plays a key role in the repair of DNA damages via the homologous recombination pathway, an impairment associated with the BRCAness phenotype that may render these HGOS patients more sensitive to targeted therapy with poly(ADP-ribose) polymerase (PARP) inhibitors [11].

In the last few years, coordinated programs and multi-institutional pipelines have been developed to translate the NGS-generated body of information into clinical applications for several tumors, including HGOS. For example, the individualized therapy for relapsed malignancies in childhood (INFORM) program (http://pediatric-neurooncology.dkfz.de/index.php/en/diagnostics/inform) is a multicentric study, the objective of which is the identification of therapeutic targets for children with malignant tumors, including HGOS [24]. The therapeutically applicable research to generate effective treatments (TARGET) project (ClinicalTrials.gov identifier: NCT01190943; http://ocg.cancer.gov/programs/target/projects/osteosarcoma) aims for the identification of genetic and epigenetic alterations of possible clinical relevance in HGOS through a combination of different genomic approaches. Recently, another strategy, consisting of the integration of genomic copy number variations and chemotherapy response-related biomarkers, has provided insights that may be of help to tailor therapy in pediatric patients with bone and soft-tissue sarcomas on the basis of individual genomic data [25].

#### 2.2.4. Epigenetics 

In addition to changes in DNA sequence, heritable epigenetic alterations targeting the whole genome can also modulate the expression of many genes, including those involved in important biologic processes such as genomic stability, DNA repair, cell cycle control and apoptosis, among others [26].

Like for other human tumors, several evidence have reported the impact of epigenetic regulation on gene expression also in HGOS [27,28]. In particular, promoter hypermethylation was indicated as the main cause for the decreased expression of 384 genes which are physiologically down-regulated in human embryonic stem cells, suggesting that this mechanism may be a means through which HGOS cells dedifferentiate and become similar to the pluripotent stem cells of their normal tissue of origin [29]. A quantitative assessment of promoter hypermethylation in multiple genes was performed in 30 paired samples of HGOS and normal tissues, revealing a significantly higher incidence of hypermethylation for several gene promoters in tumors compared to normal tissues [30]. The genes that showed the most consistent differences in promoter hypermethylation were the TIMP metallopeptidase inhibitor 3 (TIMP3), death-associated protein kinase 1 (DAPK1) and O-6-methylguanine DNA methyltransferase (MGMT). The downregulation of TIMP3 and DAPK1 genes may impact the resistance to apoptosis or tumor cell invasion [31,32,33], whereas that of MGMT may negatively affect the efficiency of DNA repair [34]. Other evidence indicated that the promoter hypermethylation of the Ras association domain family 1A (RASSF1A) gene, a tumor-suppressor gene involved in cell cycle arrest, microtubule stabilization and apoptosis, is found to be higher in HGOS, compared with normal tissues [30]. The expression of the APC down-regulated 1 (APCDD1) gene, an inhibitor of the Wnt pathway that is frequently activated in HGOS, was also demonstrated to be down-regulated through hypermethylation of its promoter in HGOS, with a consequent enhancement of tumor invasion and metastatic properties [35]. Finally, promoter hypermethylation of the cyclin-dependent kinase Inhibitor 2A (CDKN2A) gene, which acts as tumor-suppressor by regulating cell cycles through encoding the p16INK4a and p14ARF proteins, has also been reported in HGOS and suggested to be correlated with metastasis development [30].

Despite the loss of function of the TP53 and RB1 tumor-suppressor genes in HGOS that are mostly a consequence of genetic alterations, it has been reported that the expression of these genes can be dysregulated also through epigenetic mechanisms [28]. Moreover, recent findings have revealed promoter hypermethylation of the genes belonging to the TP53 pathway in HGOS experimental models and clinical samples, indicating that this epigenetic mechanism may induce the impairment of TP53-related activities [28]. In another study, down-regulation of the p14ARF protein (which inhibits MDM2, thus influencing TP53 function), as a consequence of the p14ARF gene promoter hypermethylation, was detected in 15 out of 32 HGOS clinical samples and proved to be associated with a poor survival [36]. Some authors suggested that the impact of TP53 mutations in the development of at least a subset of HGOS may be complemented by the reduced expression and loss of function of the HIC ZBTB transcriptional repressor 1 (HIC1) gene, consequent to the hypermethylation of its promoter, which was detected in the different series of the HGOS clinical samples [37,38].

The hypermethylation of the RB1 gene promoter has also been detected in HGOS, but its relevance for RB1’s loss of function remains to be established [27,39,40]. On the other hand, promoter hypermethylation of genes belonging to the RB1 pathway has been suggested to be involved in the decrease in RB1-related functions [27,40] Indeed, two studies revealed hypermethylation of the p16INK4a gene promoter, which was associated with a negative or decreased p16INK4a expression, and may impair the RB1-mediated control of cell cycles and tumor cell growth in a minority of HGOS [41,42,43,44].

Gene expression is also regulated by different RNA subclasses of non-coding RNAs, which include small non-coding RNAs (maximum length of 200 base pairs), long non-coding RNAs (lncRNAs, with length greater than 200 base pairs) and circular RNAs (small closed circular RNAs). The majority of data collected so far in HGOS concerns microRNAs (miRNAs) and lncRNAs. For details please refer to recent reviews [45,46,47]. Although the expression patterns and clinical impact of miRNAs and lncRNAs in HGOS largely need to be confirmed and better defined, on the basis of the findings reported so far, it is possible to consider them as novel attractive candidate biomarkers for monitoring disease progression, predicting outcomes and as novel candidate therapeutic targets which might be taken into consideration for planning innovative strategies of therapeutic intervention. Indeed, epigenetic regulators (first of all non-coding RNAs) were found to be involved in the reversion of tumor cells’ malignancy and the induction of cell differentiation [48]. Differentiation-based therapies would be highly desirable for tumors resistant to traditional treatments and could be a promising option for HGOS therapy, considering that the induction of differentiation in HGOS is expected to revert malignancy toward a low-grade osteosarcoma phenotype, but also to reduce tumor cells’ proliferation activity. Recently, it has been demonstrated that the inhibition of DNA methyltransferases (DNMTs), which regulate the process of DNA methylation [49], by novel nonnucleoside inhibitors, affected HGOS tumor proliferation and induced osteoblastic differentiation through the specific re-expression of genes regulating this physiologic process [50,51]. Although differentiated cells, being not or less proliferating, are thought to be less sensitive to cytotoxic drugs, Manara et al. [50] demonstrated that the novel nonnucleoside DNMT inhibitor MC3343 increased the stable doxorubicin bonds to DNA, and combined treatment resulted in sustained DNA damage and increased cell death, thus indicating a potential therapeutic option for patients with HGOS who respond poorly to neoadjuvant chemotherapy.

#### 2.2.5. Immunotherapy and Tumor Mutational Burden

Genomic research in HGOS may also provide relevant information for immunotherapeutic approaches. Immunotherapy has generated many expectancies for increasing cure probability in several cancers but, unfortunately, preclinical and clinical studies using immune checkpoint blockades led to limited and unsatisfactory results in HGOS. Several investigations have been performed or are still ongoing in order to find possible explanations for the reduced efficacy of immunotherapy in HGOS (for a review see [52]). NGS analysis of resected tumor tissues revealed an amplification of PD-L1 and PD-L2, which is associated in the literature with responses to anti-PD-1/PD-L1 blockades in other tumor types and in osteosarcoma mouse models [53]. However, data reporting on the level of the PD-1/PD-L1 expression on neoplastic HGOS cells in paraffin-embedded samples are extremely variable [54,55,56,57]. In general, the PD-L1 expression in HGOS was reported to be present only in a minority of tumor cells, partly explaining the reduced efficacy of immuno checkpoint blockade ICB in HGOS.

In addition, bone sarcomas generally exhibit a low tumor mutational burden (TMB), a predictive biomarker of anti-PD-1/PD-L1 immunotherapy responses [58,59]. Although genetic aberrations are frequent in HGOS, its TMB can be defined as low to moderate, with an average of only seven (or little more) neoepitopes per tumor [60,61], a number that, very likely, is not sufficient to efficiently stimulate the immune system and to determine a good response to anti-PD-1/PD-L1 treatments. Successful immunotherapy based on PD-1/PD-L1 blockades has so far been associated with a high TMB, which facilitates the expression of neoantigens initiating antigen-specific immune responses following immune checkpoint blockade treatment [52,58,62], and with the expression of immune checkpoint molecules. The absence of these conditions in HGOS may well explain the poor effectiveness of ICB in HGOS, supporting the need for more intense studies for a clear identification of the immune infiltrate of these tumors before scheduling the introduction of immunotherapy.

## 3. Chondrosarcoma

Chondrosarcomas are malignant mesenchymal tumors of bone which produce a cartilaginous matrix. They are the second most common primary malignant bone tumors and usually affect adults with peak incidences in the fifth to seventh decades of life. This category comprises different entities, ranging from locally-aggressive to high-grade malignant neoplasms (for a review see [63,64]. The vast majority (around 90%) are central and secondary peripheral chondrosarcomas, either presenting as de novo in the medulla of bone, or arising as a secondary tumor from pre-existing benign lesions such as enchondromas in enchondromatosis and osteochondromas. The remaining 10% are rare variants of chondrosarcomas, which include periosteal, clear cell, mesenchymal and synovial chondrosarcomas.

Although not mandatory for the diagnosis, genetic alterations characteristic of specific histotypes can be useful in the differential diagnosis with other neoplasms, in particular chondroblastic osteosarcoma. Moreover, the functional study of these alterations can help in understanding the pathogenesis of chondrosarcomas and in the design of a new specific therapy, an urgent unmet medical need, considering that chondrosarcomas are generally not responsive to standard chemotherapy or radiation therapy [65].

Genetically, two main groups of chondrosarcomas exist: central chondrosarcomas, characterized by mutations in the isocitrate dehydrogenase genes IDH1 and IDH2, and secondary peripheral chondrosarcomas, characterized by alterations in the exostosin glycosyltransferase 1 (EXT1) and 2 (EXT2) genes.

### 3.1. Central Chondrosarcomas

The central atypical cartilaginous tumor/chondrosarcoma grade 1 (ACT/CS1) is a locally aggressive hyaline cartilage-producing tumor arising in the medullary bone. Genetically, primary ACT/CS1 is characterized by somatic mutations in IDH1 and IDH2. These mutations are present in about 50% of cases. [66]. Hotspot mutations are present at the IDH1 p.Arg132 and the IDH2 p.Arg172 positions, the former being the most frequent. Mutations at the IDH1 p.Arg140 position have also been reported [67]. Grade 2 and 3 central chondrosarcomas are intermediate- and high-grade malignant tumors, respectively. Similarly, to ACT/CS1, about 50% of cases show IDH1 or IDH2 mutations. The incidence was found to be higher, up to 80%, in patients with Ollier disease and Maffucci syndrome [66], rare bone diseases characterized by multiple enchondromatosis and an increased risk of malignancies, including pancreatic adenocarcinoma, brain glioma and chondrosarcoma [68]. IDH is an enzyme of the Krebs’s cycle which catalyzes isocitrate to **α**-ketoglutarate. Somatic mutations in the IDH genes (IDH1 and IDH2) were first discovered in human glioblastomas and are associated with better overall survival [69]. Recently, hotspot sequencing of the IDH1 and IDH2 genes in 89 central chondrosarcomas followed by targeted NGS in 54 of them indicated that IDH1/IDH2 mutations are not associated with the overall survival of patients [70]. However, IDH1/IDH2 mutations were found to be associated with longer relapse-free survival and metastasis-free survival in high-grade chondrosarcomas [70].

Grade 2 and 3 central chondrosarcomas, including dedifferentiated chondrosarcomas are also characterized by complex karyotypes with aneuploidy [39,71]. Other genetic alterations described in the literature are alterations of the RB1 pathway (86% of cases) with a loss of p16 and an overexpression and/or amplification of CDK4, a mutation of TP53 (in up to 59% of the cases) [72] and mutations in COL2A1 (in 45% of cases) [73,74], and also mutations in YEATS2 (12%), EGFR (19%), NRAS (12%), and IHH signaling (18%) [67,74,75]. In about 75% of cases, CDKN2A copy number variations are present. These alterations are absent in enchondroma and ACT/CS1 [76].

**Figure 2:**
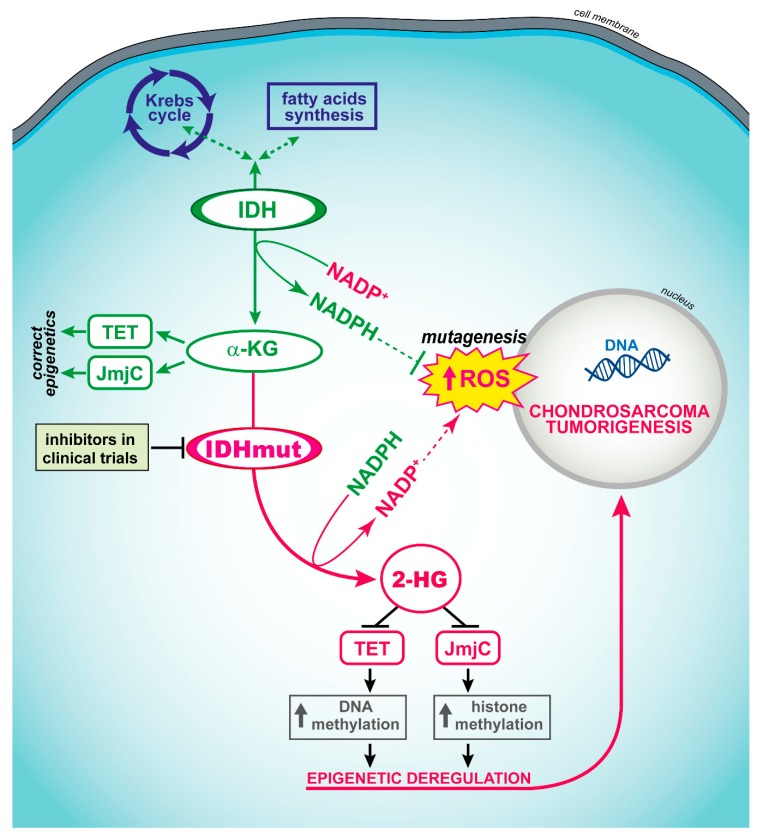
Role of isocitrate dehydrogenase (IDH) and IDH-mutated forms on the inhibition of demethylation pathways and its possible impact on chondrosarcoma genesis. Instead of isocitrate being converted to α-ketoglutarate (α-KG) with the production of reduced nicotinamide adenine dinucleotide phosphate (NADPH), α-KG is converted to 2-hydroxyglutarate (2-HG) with the consumption of reduced nicotinamide adenine dinucleotide phosphate NADPH. 2-HG is expressed at high levels in tumor cells and supports tumorigenesis by modulating Jumonji-C (JmjC)-mediated histone methylation and Tet methylcytosine dioxygenase TET-mediated DNA methylation. ROS, reactive oxygen species.

Dedifferentiated chondrosarcoma is the most aggressive entity of this group. Genetically, it is characterized by mutations in the IDH1 or IDH2 genes in 50–87% of cases. These alterations are evident in both the areas of conventional chondrosarcoma and in the high-grade dedifferentiated component [77]. IDH1 and IDH2 active site mutations result in the loss of the production of α-ketoglutarate (α-KG) and an accumulation of 2-hydroxyglutarate (2-HG) at supraphysiological levels within cells [78]. An elevated 2-HG level competitively inhibits histone lysine demethylases [79] and the TET family of 5-methylcytosine hydroxylase [80], which results in genome-wide histones and DNA methylation alterations (Figure 2). Thus, mutations in IDH1 and IDH2 may change the expression of potentially large numbers of genes, contributing to tumorigenesis through the alteration of the epigenetic control of gene expression in the cell of origin.

Selective IDH inhibitors which suppress 2-HG production and induce antitumor responses in cells with IDH1 and IDH2 mutations were developed and validated in preclinical settings. Inhibitors of mutated IDH1/2 enzymes (Figure 2) entered clinical trials for targeted therapy of gliomas [81] and may represent an interesting opportunity also for patients with chondrosarcomas. Death is rare in low-grade chondrosarcomas, while it occurs in 30% and 60% of central grade II and grade III tumors, respectively [64], encouraging claims for novel treatment strategies. Amplification of the receptor tyrosine kinases such as IGF1R and KIT which might potentially be targetable by tyrosine kinase inhibitors were also found in chondrosarcoma [70]. In addition, other pathways which might be targets for specific treatments are Hedgehog [82], mTOR [83], SRC and AKT [84]. Some targeted clinical trials are presently ongoing in chondrosarcomas (Table 2). Additional potential targets for therapy in mesenchymal, clear cell and dedifferentiated chondrosarcomas include the Bcl-2 family members and TGFβ genes as potential targets [85]. In particular, the Bcl-2 family seems to play a role in the chemoresistance of chondrosarcomas [86].

### 3.2. Secondary Peripheral Chondrosarcomas

The secondary peripheral atypical cartilaginous tumor/chondrosarcoma grade 1 (ACT/CS1) is a locally aggressive neoplasm arising within the cartilaginous cap of an osteochondroma. While in osteochondroma, EXT1 or EXT2 are biallelically inactivated in at least a subset of the tumor cells [87], the cartilaginous cap of secondary peripheral ACT/CS1, in tumor progression, becomes gradually populated by cells with at least one functional copy of EXT1 or EXT2 [88], so that EXT-mutant alleles and EXT-wildtype alleles coexist [74]. The proportion of EXT1- or EXT2-mutated alleles among all EXT alleles is about 40%. This supports the hypothesis that genetic factors other than EXT1 or EXT2 mutations, such as CDKN2A [89], are involved in tumor progression. Nevertheless, the loss of heterozygosity in the EXT1 and EXT2 genes has been implicated to cause hereditary multiple exostoses, one of the most common inherited musculoskeletal conditions, with an incidence of 1 in 50,000, whose most serious complication is chondrosarcoma transformation. The presence of germline mutations in the EXT1 or EXT2 genes in patients with multiple osteochondromatosis leads to a 5% risk of developing a secondary peripheral ACT/CS1, compared with only 1% for patients with solitary osteochondromas [90]. EXT is involved in heparan sulphate biosynthesis and the resulting heparan sulphate proteoglycans (HSPGs) are important for cell signaling. In osteochondromas in which the EXT is inactivated, the HSPGs seem to accumulate in the cytoplasm of the cell instead of being transported to be expressed at the cell surface [91], hindering the signaling pathways that are normally operative during skeletal growth, such as the Hedgehog, Wnt and TGF-β signaling pathways (for details see the review [63]). In grade 2 and grade 3 secondary peripheral chondrosarcomas, the EXT-wildtype cells seem to predominate [88]. These tumors also show more complex karyotypes and chromosomal instability. Alterations in the p53 and RB1 pathways have been described [89,92].

### 3.3. Rare Variants of Chondrosarcoma 

Periosteal chondrosarcoma is a rare malignant cartilaginous tumor arising from the bone surface. A subset of these tumors shows IDH1 and IDH2 mutations [66]. Deregulation of the RB1 signaling by the loss of the p16 expression and loss of the Wnt signaling have been described [93].

Clear cell chondrosarcoma is a rare low-grade malignant cartilaginous epiphyseal neoplasm. Clonal abnormalities with diploid or near-diploid complements, losses or structural aberrations of chromosome 9 and gains of chromosome 20 [94], CDKN2A alterations [95] and p53 overexpressions in the absence of detectable mutations [96] have been described. Interestingly, 1/15 clear cell chondrosarcomas investigated for the H3.3 mutations harbor the H3-3B (H3F3B) and p.Lys36Met mutations, suggesting a possible pathogenetic link with chondroblastoma [97].

Mesenchymal chondrosarcoma is a rare high-grade malignant tumor composed of well-differentiated hyaline cartilage and primitive undifferentiated round cells. It is characterized by a specific gene fusion between HEY1 and NCOA2 that occurs in nearly all cases [98]. Since it is absent in other morphologically similar lesions, the molecular detection of this fusion gene is important when differential diagnosis with other neoplasms should be performed. This is particularly relevant in the differential diagnosis with other round cell sarcomas when examining small biopsies of mesenchymal chondrosarcomas lacking the cartilaginous component. A single case report described a case with a IRF2BP2-CDX1 t(1;5)(q42;q32) fusion [99]. Downregulation of the RB1 pathway has also been reported [72].

Finally, synovial chondrosarcoma is an extremely rare malignant tumor, considered the malignant counterpart of synovial chondromatosis. Both these entities are characterized by FN1-ACVR2A and ACVR2A-FN1 fusions [100], which are present in at least 50% of synovial chondromatosis. Since they are present in both benign and malignant forms, the detection of these chimera cannot be used to discriminate between them.

Overall, mutations in IDH1/IDH2 or EXT1/EXT2 have been shown to have a role in the pathogenesis of most common central or peripheral chondrosarcomas, respectively. In addition, inactivation of the Rb and/or p53 pathways are present in most of the tumors and are very likely to have a major role in chondrosarcoma development. More sporadic information indicated that some genes and pathways which are involved in normal cartilage development are disrupted in the chondrosarcoma development. However, only a limited number of studies are available on chondrosarcomas, and for the rare chondrosarcoma subtypes, most genetic studies are merely restricted to case reports.

Research focusing on the elucidation of the molecular events that underlie the pathogenesis of this rare bone malignancy and the identification of new molecularly targeted therapies, especially for chemotherapy refractory chondrosarcomas, is highly desirable.

## 4. Ewing Sarcoma

Ewing sarcoma, the second most common bone sarcoma in children, is the prototype of sarcomas with simple genetic alterations. It arises more frequently in bone than soft tissues, with a small higher incidence in males compared to females. Histologically, it is characterized by a proliferation of uniform round cells.

The tumor is genetically well-characterized. Its main driver alteration is a specific chromosomal translocation that fuses a member of the FET family of proteins (encoded by FUS, EWSR1 and TAF15), which are RNA-binding proteins involved in transcription and splicing, with different members of the ETS (E26-specific) family of transcription factors, which are involved in cell proliferation, cell differentiation, cell cycle control, angiogenesis and apoptosis. EWS-FLI is the most common chimera (85% of cases) [101]. In the 15–20% of Ewing sarcomas that are negative for EWSR1–FLI1, variant fusions between EWSR1 (or rarely FUS gene) and other members of the ETS family occur, most commonly ERG (encoding transcriptional regulator ERG) followed by ETV1, ETV4, FEV and E1AF [102,103,104,105]. The detection of this specific molecular feature has been integrated in the diagnosis since the 1990s [106]. For details, see the review of this issue from Salguero-Aranda et al. (submitted to journal Cells).

Ewing sarcomas, as other translocation-associated sarcomas and many leukemias, tend to arise de novo, without a clear history of progression. Despite that 8–10% of pediatric cancers are due to germline or mosaic mutations in genes causing cancer predisposition syndromes [107], children at risk for Ewing sarcoma are not clearly defined. EWS is rarely observed in the, approximately, 120 cancer predisposition syndromes described to date [108]. Two recent genome-wide association studies suggest that the interactions between germline variation and somatically acquired EWSR1-FLI1 translocations are important etiologic contributors to EWS risk [109].

The value of the fusion chimera EWS-ets in the genesis of Ewing sarcoma has been widely established. The fusion gene is present at initiation, and it is retained throughout the evolution of the tumor. The chimeric protein is known to cause the transcriptional dysregulation of a relevant number of target genes by inducing de novo enhancers at the GGAA microsatellites (Ewing-specific enhancers) and by repressing enhancers that are active in many cell types, including putative mesenchymal cells of origin [109,110,111]. For a comprehensive review, please refer to [112]. In general, the hybrid product, primarily EWS-FLI, leads to a general remodeling of the cell transcriptome that creates a unique epigenetic signature. The chimera orchestrates multiple oncogenic hits directed toward the disruption of the normal developmental processes and causes the transformation of the cell of origin. However, despite being a necessary condition, the hybrid product is not sufficient to generate a fully transformed phenotype and requires secondary alterations, which may include mutations of the STAG2 (Cohesin subunit SA2) and TP53 genes, which are detected at diagnosis in 15–21% and 5–7% of cases, as well as a deletion of CDKN2A, a cyclin-dependent kinase that regulates cell proliferation, in 10–22% of cases, respectively [113]. Moreover, the presence of an intact IGF pathway as well as of CD99, a 32 kD integral membrane glycoprotein that is peculiarly and highly expressed in Ewing sarcoma cells, is also required for an EWS-FLI transformation [114,115].

Since there are no available drugs that can directly target the fusion proteins, researchers have tackled their downstream pathways and regulatory networks to find alternative targeted therapeutic interventions. This, also in consideration of the higher metastatic potential of EWS cells with a partial silencing of EWS-FLI [116], undermines the rationale for the complete therapeutic suppression of fusion oncoprotein transcripts.

Tumorigenesis in translocation-driven tumors is critically mediated via the IGF-1R signaling pathway [117]. Preclinical models of Ewing sarcomas show an autocrine activation of the IGF-1R-mediated signaling pathway [118], which promotes cell viability and drug escape [119,120]. Over the years, monoclonal antibodies and tyrosine kinase inhibitors were designed to specifically target IGF-1R, and several phase I to III clinical trials were conducted. From these studies, we obtained some important indications: (1) anti-IGF-1R drugs have modest toxic effects and (2) anti-IGF-1R drugs show limited effectiveness on tumors. Due to this disappointing evidence in the big-killer types of cancer, the development of anti-IGF-1R agents was largely abandoned. However, in the Ewing sarcoma clinical studies, a complete response (CR) in a few patients, a partial response (PR) in 2–12% of patients and disease stabilization in 16% to 40% patients were highlighted [121]; [122,123,124]. To date, this is one of the best results obtained in Ewing sarcomas with targeted agents. The combination of IGF-1R inhibitors and other targeted agents (for a review see [125]) or non-specific drugs such as Trabectedin have been proposed. However, despite addressing that the IGF system is still of interest in Ewing sarcomas, there are no recruiting clinical trials today. Efforts aimed to establish predictive response markers are essential for providing new fuel to the field. Besides the evaluation of IGF-1, IGF-2 and IGF-1R levels, the detection of the RNA-binding protein IGF-2-BP3 has been recently suggested [126].

Being an essential molecule for the malignancy of Ewing sarcoma, CD99 is another molecule being studied as a potential target for the treatment of this tumor. CD99 can be easily targeted by antibodies, and the availability of a human bivalent antigen-binding antibody directed against CD99 (dAbd C7) [127] that can efficiently deliver a cell death message in Ewing sarcoma cells while sparing normal cells [128] opens new therapeutic perspectives. Anti-CD99 antibodies exert additive/synergistic effects when combined with conventional agents, such as doxorubicin or vincristine [129], and are effective even against chemoresistant tumor cells. For more details, please refer to the recent reviews [130,131]. Despite these potentialities, the antibodies directed against CD99 have not reached the clinics yet. Recently, Çelik et al. described that the small molecule clofarabine was able to bind to the extracellular portion of CD99, inhibiting the biological properties of Ewing sarcoma cells both in vitro and in vivo [132], thus suggesting a targeted use of an already developed drug.

Developmental pathways and epigenetic modifiers are also of specific interest in fusion-driven sarcomas. Ewing sarcoma is characterized by the paucity of other genetic mutations [113,133,134] and due to the functions of their disease-defining oncogenes, epigenetic dysregulation plays an important role in the maintenance of their phenotype. On this basis, several drugs targeting epigenetic modulators have been tested in preclinical conditions against Ewing sarcoma cells. In particular, drugs against the histone deacetylase (HDAC), an epigenetic regulator of histone methylation [135], the enhancer of zeste homolog 2 (EZH2), a polycomb group protein involved in DNA methylation [136], the bromodomain and extra terminal domain (BET) family, whose members are involved in chromatin regulation, and lysine-specific demethylase 1 (LSD1) have provided interesting data that led to their inclusion in clinical trials. Currently, two phase I trials are recruiting Ewing sarcoma patients to test LSD1 inhibitors (Table 3). Interestingly, the LSD1 inhibitor SP2509 was shown to display synergistic effects with the HDAC inhibitors suberoylanilide hydroxamic acid (SAHA) or romidepsin in vivo [137]. Based on these preclinical studies, more investigations focusing on combinations with different epigenetic inhibitors are warranted.

Finally, the inhibition of EWS-FLI1 binding to RNA helicase A (RHA), which is important for its oncogenic function, was shown to have therapeutic relevance [138,139], while functional, genomic and super-enhancer screenings identified a peculiar sensitivity of Ewing sarcomas to the Cyclin D1/CDK4 pathway [140], and to Poly-ADP-ribose polymerase (PARP) inhibitors [141,142]. Ewing sarcoma patients have been recruited in several trials where these agents have been used in monotherapy [143] or in combination [144,145,146,147].

A list of the presently active targeted clinical trials in Ewing sarcomas is provided in Table 3.

In general, many of these targeted therapies are expected to suffer from the development of resistance. Therefore, the identification of the most effective combination treatments and of adequate schedules may led to the design of innovative, more effective treatments. For example, Salvador-Barbero et al. have recently shown that sequential treatment with CDK4/6 inhibitors following DNA-damaging chemotherapy enhances therapeutic benefits in pancreatic cancer models, despite previous reports that concurrent treatment did not [148].

## 5. Critical Open Issues and Perspectives

Carrying out molecular studies in patients is necessary, at the best by massive analysis technologies (NGS), to find new potentially actionable molecular targets. This is certainly true for tumors that are still orphans for innovative, targeted drugs. In the era of personalized medicine, the rarity of sarcomas is not a major obstacle, provided that each patient is studied extensively.

Due to the lack of specific, druggable genetic alterations in bone sarcomas, researchers are still forced to work at a discovery level. The application of RNA-seq, a technique that likely reveals biologically informative transcriptional pathways, is the first-line indication. However, it remains unknown how deep the sequencing datasets should be performed in the challenging context of identifying transcriptional signatures that are robustly associated with disease outcome and that may indicate novel therapeutic approaches.

Personalized treatments in refractory patients cannot be effective without knowledge of risk and response. A cross-validation of the results is essential to obtain robust information and high-quality relationships, and international collaboration should be largely encouraged. Collaboration with patients should be stimulated to happen throughout the research process and researchers/clinicians should set the context for patients to contribute meaningfully.

The development of experimental models that may well reflect the biological and genetic diversity of the tumors should be encouraged as an essential step to prioritize but also de-prioritize the therapeutic agents that are raised to the attention of clinicians.

Most patients with bone tumors did not have a known cancer predisposition syndrome. However, for chondrosarcoma and osteosarcoma, a link has been observed, suggesting there might be an unknown genetic susceptibility underlying their development. Investigations of the family history and germline genetic association studies might help elucidate the potential genetic susceptibility in the future.

The possibility of genetic studies over time in the same patient, provided by the study of circulating tumors’ DNA/RNA with highly sensitive techniques, enables the study of prognostic and/or predictive biomarkers, the determination of minimal residual diseases, early detection of resistance and the study of tumor evolution. The application of these analyses will also help patients with predisposition syndromes and should be highly encouraged for pediatric patients

A percentage of patients with bone sarcomas also have histories of second malignancies that are unrelated to bone tumors. A close follow-up of the patient is necessary and appropriate. Detecting a cancer early reduces treatments and treatment-associated morbidity.

Immunotherapy approaches in bone sarcomas have led to disappointing results, so far. The application of massive and parallel analyses is required to determine the specific composition of the immune microenvironment for primary bone sarcomas. 

## 6. Concluding Remarks

The identification of cancer-specific molecular alterations and vulnerabilities is an important objective for bone sarcomas. The detection of tumor-specific molecular alterations is already into routine clinicopathological practice as a molecular tool to assist the pathologist in the differential diagnosis of several histotypes of bone sarcomas. However, a deeper systematic molecular analysis of these lesions is highly warranted to help us to better understand the pathogenesis of these tumors, and to hopefully enable the development of novel and more personalized therapeutic strategies. Early-phase clinical trials guided by comprehensive molecular tumor profiling have become feasible thanks to the reduction of costs for NGS studies and the increased robustness and standardization of procedures. Considering the rarity of these tumors, the definition of a clear, internationally approved road map that combines emerging genomic and functional approaches with a selection of novel therapeutic strategies could provide the basis for transforming the care of patients with bone sarcomas.

## Figures and Tables

**Table 1:** List of target-specific clinical trials that are presently active and recruiting high-grade osteosarcoma (HGOS) patients. Time period refers to the actual study start date and estimated study completion date.

Treatment	Mechanism of Action	Bone Sarcoma Histotypes	ClinicalTrials.gov NCT Identifier (Protocol Acronym)	Participating Countries	Stage of Development (Time Period)
**Losartan + Sunitinib**	Sunitinib: multi-target inhibition of RTK	HGOS	NCT03900793	USA	phase I (08/2019–02/2025)
**Famitinib** plus **Camrelizumab (SHR-1210) or Famitinib alone** **or Famitinib** plus **Ifosfamide**	Famitinib: multi-target inhibition of RTK, including SCFR (c-Kit), VEGFR2 and 3, PDGFR, Flt1 and Flt3 Camrelizumab: inhibition of PD-1 immune checkpoint	advanced HGOS	NCT04044378	China	phase I/II (08/2019–09/2022)
**Pazopanib hydrochloride** (Votrient^®^) with oral **Topotecan hydrochloride**	Inhibition of VEGFR-1, -2, -3, PDGFR-α and -β, and KIT (Pazopanib)	recurrent or metastatic HGOS	NCT02357810	USA	phase II (02/2015–06/2022)
**Apatinib (YN968D1)** in combination with chemotherapy	Inhibition of VEGFR2	HGOS with pulmonary metastasis	NCT03742193	China	phase II (03/2019–09/2022)
**Regorafenib** (BAY 73-4506, commercial name Stivarga)	Multi-kinase inhibitor targeting VEGFR2, TIE2, PDGFR-beta, FGFR, KIT, RET, and RAF	HGOS, Ewing sarcoma	NCT02048371 (SARC024)	USA	phase II (07/2014–12/2020)
**Regorafenib** (BAY 73-4506, commercial name Stivarga)	Multi-kinase inhibitor targeting VEGFR2, TIE2, PDGFR-beta, FGFR, KIT, RET, and RAF	metastatic bone sarcomas (HGOS, Ewing sarcoma, chondrosarcoma)	NCT02389244 (REGOBONE)	France	phase II (09/2014–03/2023)
**Cabozantinib-S-Malate** (Cabometyx; Cometriq)	Inhibition of MET, VEGFR2, AXL and RET	recurrent, refractory, or newly diagnosed sarcomas, including HGOS	NCT02867592	USA	phase II (05/2017–06/2020)
**Erdafitinib (JNJ-42756493)**	FGFR inhibitor with negative effects on angiogenesis	relapsed or refractory advanced solid tumors, including HGOS	NCT03210714 (The Pediatric MATCH Screening Trial)	USA	phase II (11/2017–12/2024)
**Palbociclib** (PD-0332991, trade name Ibrance)	Selective inhibition of the cyclin-dependent kinases CDK4 and CDK6	relapsed or refractory advanced solid tumors, including refractory HGOS	NCT03526250 (The Pediatric MATCH Screening Trial)	USA	phase II (06/2018–06/2025)
**Abemaciclib** (Verzenios)	Selective inhibition of the cyclin-dependent kinases CDK4 and CDK6	recurrent or refractory solid tumors, including HGOS and Ewing sarcoma	NCT02644460	USA	phase I (02/2016–12/2020)
**Abemaciclib** (Verzenios)	Selective inhibition of the cyclin-dependent kinases CDK4 and CDK6	advanced HGOS and chondrosarcoma	NCT04040205	USA	phase II (10/2019–09/2024)
**Samotolisib (LY3023414)**	Inhibition of PI3K/AKT/mTOR pathway	relapsed or refractory advanced solid tumors, including HGOS	NCT03213678 (The Pediatric MATCH Screening Trial)	USA	phase II (07/2017–09/2024)
**Berzosertib** (M6620; VX-970; Captisol^®^)	Selective inhibitor of ATR	HGOS	NCT03718091	USA	phase II (01/2019–04/2025)
**Olaparib** (AZD-2281, MK-7339 trade name Lynparza^®^)	Inhibition of PARP1, opposing DNA repair, in patients with hereditary BRCA1 or BRCA2 mutations	relapsed or refractory advanced solid tumors, including HGOS	NCT03233204 (The Pediatric MATCH Screening Trial)	USA	phase II (07/2017–09/2024)
**Ulixertinib (BVD-523; VRT752271)**	Inhibition of ERK1/2 kinases, belonging to the MAPK pathway	relapsed or refractory advanced solid tumors, including recurrent HGOS	NCT03698994 (The Pediatric MATCH Screening Trial)	USA	phase II (10/2018–12/2025)
**Vemurafenib** (PLX40321; Zelboraf^®^)	Inhibition of the mutated B-Raf protein, interrupting its stimulation of cell growth	relapsed or refractory advanced solid tumors, including HGOS	NCT03220035 (The Pediatric MATCH Screening Trial)	USA	phase II (07/2017–12/2023)
**Larotrectinib** (ARRY-470; LOXO-101; Vitrakvi^®^)	Inhibition of tropomyosin kinase receptors TrkA, TrkB, and TrkC	relapsed or refractory advanced solid tumors, including HGOS	NCT03213704	USA	phase II (07/2017–09/2024)
**9-ING-41** with **Gemcitabine, Doxorubicin, Lomustine, Carboplatin, Nab paclitaxel, Paclitaxel**	9-ING-41: inhibition of GSK-3	advanced cancers, including HGOS	NCT03678883	USA	phase I/II (01/2019–11/2022)
**Tazemetostat** (EPZ-6438)	Inhibition of the activity of human polycomb repressive complex 2 -containing wild-type histone-lysine N-methyltransferases EZH1 and EZH2	advanced cancers, including HGOS and Ewing sarcoma	NCT03213665 (The Pediatric MATCH Screening Trial)	USA	phase II (07/2017–09/2024)
**Avelumab (Bavencio^®^)**	Targeting PD-L1	recurrent or progressive HGOS	NCT03006848	USA	phase II (02/2017–01/2023)
**ZKAB001** (STI-1014; STI-A1014)	Targeting PD-L1	recurrent or refractory HGOS	NCT03676985	China	phase I/II (10/2018–06/2023)
**Nivolumab (Opdivo^®^)** with or without **Azacitidine**	Targeting PD-1 (Nivolumab)	recurrent HGOS	NCT03628209	USA	phase I/II (07/2019–07/2022)
**Pembrolizumab (MK3475)** combined with **metronomic Cyclophosphamide**	Targeting PD-1 in association with metronomic chemotherapy	advanced sarcomas	NCT02406781	France	phase II (06/2015–06/2023)
**Pepinemab** (VX15/2503)	Targeting Semaphorin-4D (SEMA4D), also known as Cluster of Differentiation 100 (CD100), which binds to CD72 to activate B cells and dendritic cells	recurrent and refractory HGOS	NCT03320330	USA	phase I/II (01/2018–09/2021)
**4th generation safety-engineered CAR T cells targeting sarcomas**	4th generation safety-engineered CAR T cells targeting sarcoma surface antigens	sarcomas including HGOS and Ewing sarcoma	NCT03356782	China	phase I (12/2017–12/2020)
**EGFR806 CAR T cell immunotherapy**	second generation EGFR-specific CAR T cells, which have been genetically modified to express either the EGFR receptor alone (EGFR806CAR(2G)-EGFRt) or in addition also the CD19 receptor (CD19CAR(2G)-T2A-HER2tG)	recurrent or refractory solid tumors, including HGOS and Ewing sarcoma	NCT03618381	USA	phase I (06/2019–06/2036)
**C7R-GD2.CAR T cell immunotherapy**		relapsed or refractory GD2-positive tumors, including HGOS and Ewing sarcoma	NCT03635632	USA	phase I (04/2019–12/2037)
**Humanized Monoclonal Antibody 3F8 (Hu3F8)** combined with GM-CSF	Targeting GD2 with the humanized antibody Hu3F8	recurrent HGOS	NCT02502786	USA	phase II (07/2015–07/2021)
**Humanized anti-GD2 bispecific antibody Hu3F8-BsAb**	Targeting GD2 with the bispecific antibody Hu3F8-BsAb	HGOS	NCT03860207	USA	phase I/II (02/2019–02/2022)

Legend: ATR, ataxia telangiectasia and rad3-related; BRCA, breast related cancer antigen; CD100, cluster of differentiation 100; CDK, cyclin-dependent kinase; EGFR epidermal growth factor receptor; ERK, extracellular signal–regulated kinase; Flt, FMS-like tyrosine kinase; FGFR, fibroblast growth factor receptor; GD2, disialoganglioside 2; GSK-3, glycogen sinthase kinase 3; HGOS, high-grade osteosarcoma; mTOR, mammalian target of rapamycin; MAPK, mitogen-activated protein kinase; PDGFR, platelet-derived growth factor receptor; PARP1, poly (ADP-ribose) polymerase 1; PI3K, phosphatidylinositol 3-kinase; RAF, rapidly accelerated fibrosarcoma; PD-1, programmed cell death 1; PD-L1, programmed death-ligand 1; SCFR (c-Kit) stem cell factor receptor; RET, rearranged during transfection; RTK, receptor tyrosine kinase; SEMA4D, semaphorin-4D; TRK, tropomyosin kinase receptor; TIE2, tyrosine kinase with immunoglobulin-like and EGF-like domains 2; VEGFR, vascular endothelial growth factor receptor.

**Table 2:** List of target-specific clinical trials that are presently active and recruiting chondrosarcoma patients. Trials that are also recruiting high-grade osteosarcoma patients are listed in Table 1. Time period refer to the actual study start date and estimated study completion date.

Treatment	Mechanism of Action	Bone Sarcoma Histotypes	ClinicalTrials.gov NCT Identifier (Protocol Acronym)	Participating Countries	Stage of Development (Time Period)
**Sirolimus** and **Cyclophosphamide**	Inhibition of mTOR signalling	Conventional, Mesenchymal and Dedifferentiated Chondrosarcomas	NCT02821507 (COSYMO)	Netherlands Spain	phase II (06/2014–06/2021)
**FT 2102 (Olutasidenib)**	Inhibitor of mutant IDH1	Chondrosarcoma	NCT03684811	USA	phase II (11/2018–04/2022)

Legend: IDH1, Isocitrate dehydrogenase 1; mTOR, mammalian target of rapamycin.

**Table 3:** List of target-specific clinical trials that are presently active and recruiting Ewing sarcoma patients. Trials that are also recruiting high-grade osteosarcoma or chondrosarcoma patients are listed in Table 1 and Table 2. Time period refers to the actual study start date and estimated study completion date.

Treatment	Mechanism of Action	Bone Sarcoma Histotypes	ClinicalTrials.gov NCT Identifier (Protocol Acronym)	Participating Countries	Stage of Development (Time Period)
**SP-2577 (Seclidemstat)**	Inhibition of the LSD1 epigenetic enzyme	Ewing Sarcoma	NCT03600649	USA	phase I (06/2018–12/2021)
**INCB059872**	Inhibition of the LSD1 epigenetic enzyme	relapsed or refractory Ewing sarcoma	NCT03514407	USA Italy Spain UK	phase I (06/2018–06/2021)
**Niraparib** and **Temozolomide** and/or **Irinotecan**	Niraparib: inhibition of PARP	previously treated, incurable Ewing sarcoma	NCT02044120	USA UK	phase I (05/2014–04/2021)
**Pbi-shRNA™ EWS/FLI1 Type 1 LPX**	Targeting EWS/FLI1 type 1 fusion transcript	advanced Ewing sarcoma	NCT02736565	USA	phase I (10/2016–02/2022)
**TK216**	Inhibition of the between EWS-FLI1 and RNA helicase A through binding to EWS-FLI1	relapsed or refractory Ewing sarcoma	NCT02657005	USA	phase I (08/2016–06/2021)
**Vorinostat (Zolinza)** in combination with chemotherapy	Inhibition of HDAC	Ewing Sarcoma	NCT04308330	USA	phase I (03/2017–12/2022)
**Olaparib** and Temozolomide	Inhibition of PARP	Ewing Sarcoma	NCT01858168	USA	phase I (07/2013–07/2024)
**Anlotinib** and **Irinotecan**	Multi-target inhibition of RTK, including VEGFR2 and VEGFR3	metastatic Ewing Sarcoma	NCT03416517	China	phase I (01/2018–12/2020)
**Palbociclib** combined with chemotherapy	Inhibition of CDK4/6	Ewing Sarcoma	NCT03709680	USA	phase I (05/2019–04/2024)
**Palbociclib** plus **Ganitumab**	Palbociclib: inhibition of CDK4/6 Ganitumab: inhibition of IGF-1R	Ewing Sarcoma	NCT04129151	USA	phase II (12/2019–08/2022)
**Eribulin Mesylate**	Inhibition polymerization of tubulin subunits impairing the EWS-FLI1 mediated microtubule stabilization	Ewing Sarcoma	NCT03441360	USA	phase II (04/2018–06/2020)
**Eribulin Mesylate**	Inhibition polymerization of tubulin subunits impairing the EWS-FLI1 mediated microtubule stabilization	Ewing Sarcoma	NCT03245450	France Germany Greece Italy Spain UK	phase II (08/2017–09/2021)
**Nivolumab (Opdivo^®^)** plus **ABI-009 (Nab-rapamycin; Nab-sirolimus)**	Targeting PD-1 (Nivolumab) and mTOR (ABI-009)	Ewing sarcoma	NCT03190174	USA	phase I/II (08/2017–04/2021)

Legend: CDK, cyclin dependent kinase; EWS-FLI1, Ewing Sarcoma-Friend Leukemia Insertion; HDAC: histone deacetylases; IGF-1, Insulin-like growth factor 1 receptor; LSD1, lysine-specific demethylase 1 enzyme; mTOR, mammalian target of rapamycin; PARP, poly (ADP-ribose) polymerase; PD-1, programmed cell death 1; RTK, receptor tyrosine kinase; VEGFR, vascular endothelial growth factor receptor.

## Abbreviations

The abbreviations including in the text are reported alphabetically.

ACT/CS1	atypical cartilaginous tumour/chondrosarcoma grade 1
ACVR2A-FN	activin receptor 2A- fibronectin
AKT	Serine/Threonine Kinase+
APC	APC regulator of WNT signaling pathway
APCDD1	APC down-regulated 1
Bcl-2	Apoptosis Regulator
BET	the bromodomain and extra terminal domain
BRCA1	breast related cancer antigen 1
BRCA2	breast related cancer antigen 2
CD99	CD99 Molecule (XgBloodGroup)
CDK4	cyclin-dependent kinase 4
CDK4/6	cyclin-dependent kinase 4/6
CDKN2A	cyclin-dependent kinase Inhibitor 2A
CR	complete response
DAPK1	death-associated protein kinase 1
DNA/RNA	DeoxyriboNucleic Acid/RiboNucleic Acid
DNMTs	DNA methyltransferases
EGFR	epidermal growth factor receptor
ERCC1	excision repair cross-complementation group 1
ERCC2	excision repair cross-complementation group 2
ERG	ETS transcription factor ERG
ETS	proto-oncogene 1, transcription factor 1
ETV1	ETS Variant Transcription Factor 1
ETV4	ETS Variant Transcription Factor 4
EWS-FLI	Ewing Sarcoma-Friend Leukemia Insertion
EWS	Ewing Sarcoma
EWSR1	Ewing sarcoma breakpoint region 1, EWS RNA Binding Protein 1
EWSR1–FLI1	Ewing sarcoma breakpoint region 1 (EWS RNA Binding Protein 1) - Friend Leukemia Insertion
EXT	exostosin glycosyltransferase
EZH2	enhancer of zeste homolog 2
FEV	FEV Transcription Factor, ETS Family Member
FUS	FUS RNA Binding Protein
GD2	disialoganglioside 2
HDAC	histone deacetylase
HEY1	Hes Related Family BHLH Transcription Factor With YRPW Motif 1
HGOS	High-grade osteosarcoma
HIC1	HIC ZBTB transcriptional repressor 1
HSPGs	heparan sulphate proteoglycans
ICB	immune checkpoint blockade
IDH	isocitrate dehydrogenase genes
IGF-1	Insulin-like growth factor 1
IGF-1R	Insulin-like growth factor 1 receptor
IGF-2	Insulin-like growth factor 2
IGF	Insulin-like growth factor
IGF-2BP3	Insulin-like growth factor 2 - mRNA-binding protein 3
IHH	Indian Hedgehog Signaling Molecule
INFORM	individualized therapy for relapsed malignancies in childhood
IRF2BP2-CDX1	Interferon Regulatory Factor 2 Binding Protein 2-Caudal Type Homeobox 1
ISG	Italian Sarcoma Group
KDR	kinase insert domain receptor
KIT	Proto-Oncogene, Receptor Tyrosine Kinase
lncRNA	Long non-coding RNA
lncRNAs	long non-coding RNAs
LSAMP	Limbic System-Associated Membrane Protein
LSD1	Lysine Demethylase 1°
MDM2	MDM2 Proto-Oncogene
MGMT	O-6-methylguanine DNA methyltransferase
miRNA	microRNA
miRNAs	microRNAs
MSH2	mut S homolog 2
MTHFR	5,10-methylenetetrahydrofolate reductase
mTOR	mammalian target of rapamycin
NADPH	reduced nicotinamide adenine dinucleotide phosphate
NCOA2	Nuclear Receptor Coactivator 2
NER	Nucleotide excision repair
NGS	Next-generation sequencing
NRAS	NRAS Proto-Oncogene
p16	also known as p16INK4a, cyclin-dependent kinase inhibitor 2A or CDKN2A, multiple tumor suppressor 1
PALB2	partner and localizer of BRCA2
PARP	poly (ADP-ribose) polymerase
PD-1	programmedcelldeath 1
PD-L1	programmed death-ligand 1
PI3K/mTOR	phosphatidylinositol 3-kinase/mammalian target of rapamycin
PR	partial response
RASSF1A	Ras association domain family 1A
RB1	RB TranscriptionalCorepressor 1
RECQL	RecQ-like helicase
RHA	RNA helicase A
SAHA	suberoylanilide hydroxamic acid
SNP	Single nucleotide polymorphism
SQSTM1	Sequestosome 1
SRC	SRC Proto-Oncogene, Non-Receptor Tyrosine Kinase
STAG2	Stromal Antigen 2
SV	structural variations
TAF15	TATA-Box Binding Protein Associated Factor 15
TARGET	Generate Effective Treatments Osteosarcoma project
TET	Tet Methylcytosine Dioxygenase
TGFβ	transforming growth factor β
TIMP3	TIMP metallopeptidase inhibitor 3
TMB	tumor mutational burden
TP53	Tumor Protein P53
WWOX	WW Domain ContainingOxidoreductase
α-KG	α-ketoglutarate
2-HG	2-hydroxyglutarate
